# A case report on congenital hypothyroidism and alpha thalassemia in children with anemia and muscle damage as the main manifestation

**DOI:** 10.1097/MD.0000000000039446

**Published:** 2024-08-16

**Authors:** Ying Zhang, Xuan Li, Xiao-jun Wang, Ju-pin Yang, Ju-mei Li, Wen-qian Yuan, Yu-ying Dong, Jin-peng Yu, Yu Wen, Ming-wei Liu

**Affiliations:** a Department of Child Rehabilitation, Dali Bai Autonomous Prefecture People’s Hospital, Dali, Yunnan, China; b Department of Rehabilitation, Dali Bai Autonomous Prefecture People’s Hospital, Dali, Yunnan, China; c Department of Medical Laboratory, Dali Bai Autonomous Prefecture People’s Hospital, Dali, Yunnan, China; d Department of Pediatrics, Dali Bai Autonomous Prefecture People’s Hospital, Dali, Yunnan, China; e Department of Emergency, The First Affiliated Hospital of Kunming Medical University, Kunming, China; f Department of Emergency, Dali Bai Autonomous Prefecture People’s Hospital, Dali, Yunnan, China.

**Keywords:** alpha thalassemia, anemia, congenital hypothyroidism, muscle damage

## Abstract

**Rationale:**

This study reports the first case of congenital hypothyroidism (CH) and alpha thalassemia in a child in China, with anemia and muscle damage as the main manifestations. Analyzing and studying this case is of great significance in reducing missed and misdiagnosed CH and will provide a clinical strategy for treating these patients.

**Patient concerns:**

Child, female, 2 years and 7 months old, the child appeared dispirited, had poor appetite, shallow complexion, reduced activities with anemia, elevated muscle enzymes, height, and growth retardation.

**Diagnoses:**

The child was diagnosed with CH with alpha thalassemia.

**Interventions:**

The patient was treated with levothyroxine sodium and anemia correction.

**Outcomes:**

The children’s current spirit, appetite, red face, normal limb activity, physical development, and intelligence were significantly better than those of normal children of the same age.

**Conclusions:**

CH with alpha thalassemia, especially anemia and muscle damage as the main manifestations, has not been reported. Administration of levothyroxine sodium is effective in correcting anemia in patients with CH and alpha thalassemia.

**Lesson:**

Due to CH and alpha thalassemia, there are no specific symptoms and they are prone to missed diagnosis and misdiagnosis. Therefore, patients with anemia and elevated muscle enzyme levels should be routinely tested for thyroid function to diagnose them early and provide proper treatment to avoid negative consequences.

## 1. Introduction

Congenital hypothyroidism (CH) is caused by insufficient generation of thyroid hormones or a congenital disease of the receptor defect in the thyroid. Its main clinical features include slow growth and mental retardation.^[1]^ The morbidity associated with CH is high in children. Because of the participation of thyroid hormones in regulating the metabolism of various systems of the whole body,^[2]^ clinical features of CH could be complex and diverse, and its symptoms could be atypical. In particular, when CH is combined with other genetic diseases, it is more likely to be misdiagnosed and its treatment delayed.

Thalassemia is caused by an α- or β-mutation or deletion of the globin gene, which causes α- or β-globin chain synthesis to be reduced or completely unable to synthesize, resulting in hereditary hemolytic diseases. Thalassemia is the most common single-gene recessive genetic disease in the world. Clinically, it is mainly divided into α-thalassemia and β-thalassemia, carried by approximately 1.5% of the world’s population with β-variation in the globin gene^[3]^ and 5% of the population with α-variation in globin.^[4]^ Thalassemia mainly occurs in the Mediterranean region, sub-Saharan Africa, the Middle East, the Indian subcontinent, East Asia, and Southeast Asia,^[5,6]^ while most areas south of the Yangtze River in China, especially Guangdong, Guangxi, and Hainan, have a high incidence of the disease.^[7]^ According to the Blue Book on Thalassemia in China (2020), there were approximately 30 million carriers of thalassemia in China in 2015, and 300,000 intermediate and severe patients in total. With an increase in global migration, land poverty has become a global health burden.^[8]^

The incidence of α-thalassemia is low in the Yunnan Province, China. However, CH with α-thalassemia, especially anemia and muscle damage as the main manifestations, has not yet been reported. Herein, we report the case of a child with CH and alpha thalassemia, with anemia and muscle damage as the main manifestations.

## 2. Case report

### 2.1. Ethics approval and consent to participate

Informed written consent was obtained from the patient and patient’s parents for the publication of this case report and accompanying images.

This study was reviewed and approved by the local Medical Ethics Committee of Dali Bai Autonomous Prefecture People’s Hospital. The procedures were performed in accordance with the Helsinki Declaration of 1975 and were revised in 2000.

### 2.2. Medical history

The female child, 2 years and 7 months, since November, 2014, the patient had a shallow complexion, anemia, poor appetite, and reduced activity for more than 2 months. Mediterranean genotyping in the local hospital showed that the children had gene deletion (types 3 and 7), α-thalassemia 2 gene heterozygote (-α/αα), nerve conduction velocity of both lower limbs: (1) the CMAP amplitude of the bilateral tibial nerves decreased, and H-reflex of the left tibial nerve did not occur. (2) The snap of the bilateral sural nerve and F wave of the right sural nerve were not extracted. Electromyography revealed no obvious abnormalities. The amplitude of the MUP at the tested part of the lower limb was normal, the duration of the MUP was normal, and the denervation potential appeared during relaxation. Cardiac color Doppler ultrasound revealed no abnormalities in heart size or ventricular wall mobility, no obvious shunt in the heart, mild regurgitation of the tricuspid and pulmonary valves, or a small amount of pericardial effusion. Abdominal color Doppler ultrasonography: (1) no obvious abnormality was found in the ultrasound images of the liver, gallbladder, pancreas, spleen, and kidney; (2) small amount of ascites. No obvious abnormalities were found on MRI. No abnormalities were found in the progressive muscular dystrophy gene detection, mitochondrial gene DNA detection, or spinal muscle atrophy gene detection. Diagnosed as α-thalassemia, moderate anemia, familial progressive muscular dystrophy, spinal muscular atrophy, mitochondrial encephalomyopathy, and congenital neuromuscular diseases. The patients were given 0.5 µl concentrated suspended red blood cells, levocarnitine, Viagra, nutritional support, and other treatments, and the symptoms were not significantly relieved. In July 2015, the child was admitted to the Department of Pediatrics of the People’s Hospital of the Dali Bai Autonomous Prefecture in Yunnan Province for treatment.

### 2.3. Past medical history

The child was born in December 2012 by cesarean section at 40 weeks of pregnancy, weighed 3600 g, and had no records of particular diseases. Her parents claimed that both her growth and intelligence were normal before one and a half years old. Her father indicated gene deletion (types 3 and 7), α-thalassemia 2 gene heterozygous (-α/αα), which was diagnosed as “alpha thalassemia”.

### 2.4. Physical examination

Body temperature 36.4 °C, pulse 110 times/min, breathing 24 times/min, height 80 cm, weight 11 kg, poor development altogether, clear mind, mental depression, poor response, intelligence rough test similar to the same age, face waxy yellow, moderate anemia appearance, no obvious special face, front 1.5 cm × 1.5 cm, slightly pale lip, no blood on the mouth, double tonsils 1° swollen, double lung breathing sound clear, unheard and wet, heart tone strong, rhythm neat, unheard and murmur, abdominal soft, liver and spleen untouched, whole abdominal no lumps, muscle tension and anti-jumping pain, quadriplegic strength, lower heel tension, lower heel muscle, muscle can walk independently, squat standing laborious, a bed slightly pale, physiological reflection exists, pathological reflection is not emitted.

### 2.5. Laboratory data

Thyroid function of local county hospitals (July 15, 2015) Tip: free triiodine thyroxine (FT3) 1.65 ng/dL, free thyroxine (FT4) 0.49 ng/dL, triiodedosine (T3) 15.77ng/dL, thyroxine (T4) 0.89 ng/dL, thyroxine (TSH) and 100mIU/L. Thyroid color super tip: the measurement value is smaller than the same age normal children. The blood routine and blood biochemical results in May 2015 are shown in Tables 2 and 3.

### 2.6. Diagnosis and treatment

**Figure 1.**
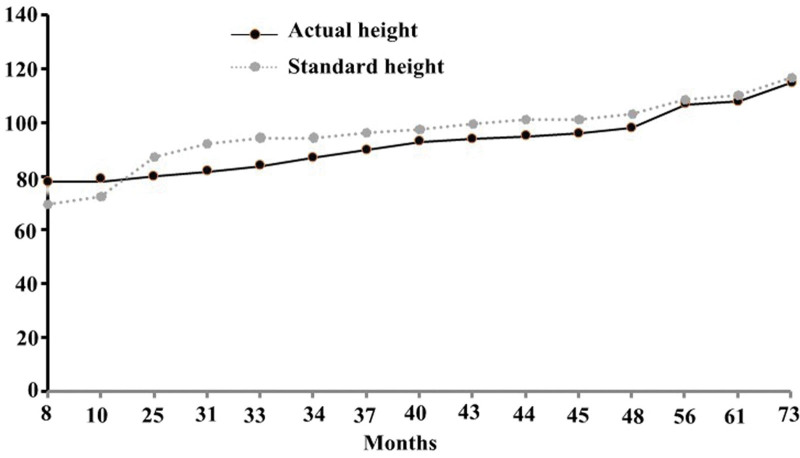
The height changes of the child in this case.

Based on the patient’s medical history, symptoms, and laboratory data, the child was diagnosed with CH, hypothyroidism, muscle damage, and alpha thalassemia (static type). The treatment was administered as an alternative to sodium hypothyroxine (euphelin) (starting at 25 µg per day and gradually increasing to 50 µg per day). After 1 month of treatment with sodium hypothyroxine, the children’s spirit, appetite, red-faced, normal limb activity, physical, and intelligence gradually improved (Tables 1–4, and Fig. 1). The patient was discharged and continued to receive regular oral levothyroxine.

**Table 1:** Changes in height and weight of this child.

Age	Heights (cm)	Standard (cm)	Weight (kg)
8 months	78	69.6	8.2
10 months	79	72.4	9
25 months	80	87.2	10
31months*	82	92.1	10
33 months	84	94.3	11
34 months	87	94.3	12
37 months	90	96.3	13
40 months	93	97.5	14
43 months	94	99.4	N/A
44 months	95	101.2	N/A
45 months	96	101.2	N/A
48 months	98	103.1	N/A
56 months	107	108.5	N/A
61 months	108	110.2	N/A
73 months	115	116.6	21

*Note*: The height standard refers to the “Reference Standards for the Growth and Development of Children Under 7 in China”.

* Treatment with Euthyrox began.

**Table 2:** Changes of hemoglobin in this case.

Date	HB (g/L)	MCV (Fl)	MCH (pg)	MCHC (g/L)
November 17, 2014	84	90.8	29.7	327
December 8, 2014	83	91.6	29.1	318
December 11, 2014	107	91.6	29.8	325
December 16, 2014	99	92.5	29.6	320
December 21, 2014	103	92.6	29.5	319
March 12, 2015	80	93.6	30.2	323
March 14, 2015	77	90.8	29.5	325
March 15, 2015	75	93.2	30.1	323
March 17, 2015*	111	92.5	29.8	322
May 25, 2015	91	90.6	29.6	327
May 27, 2015*	125	87.2	28.6	328
May 31, 2015	113	90,9	28.6	315
August 17, 2015†	89	89	29.9	337
November 10, 2015	106	82	27	329
March 1, 2016	118	75	26.1	347
June 27, 2016	102	78	24.1	310
September 7, 2016	109	79	25.3	319
November 1, 2016	118	80	25.9	325
September 3, 2017	115	80	26	325
March 28, 2018	126	79.0	27.3	347
December 30, 2018	127	79	26.8	342

HB = hemoglobin, MCH = mean corpuscular hemoglobin, MCHC = mean erythrocyte hemoglobin concentration, MCV = mean red blood cell volume.

* Blood transfusion treatment time.

† Treatment with Euthyrox began.

**Table 3:** Changes in serum muscle enzyme spectrum of this child.

Date	CK (U/L)	CK-MB (U/L)	LDH (U/L)	HBDH (U/L)	AST (U/L)
November 19, 2014	2886	59	560	505	91
December 8, 2014	2984	31	1338	N/A	158
December 16, 2014	1037	30	352	296	73
December 21, 2014	1757	45	450	373	102
March 12, 2015	1830	N/A	346.7	306	97
May 25, 2015	2536	51	1158	N/A	119
May 31, 2015	1831	33	401	375	66
August 17, 2015*	1154	27	193	154	32
September 3, 2017	110	23	191	162	30

AST = aspartate aminotransferase, CK = creatine kinase, CK-MB = creatinekinase-MB, HBDH = hydroxybutyrate dehydrogenase, LDH = lactate dehydrogenase.

* Treatment with Euthyrox began.

**Table 4:** Changes in thyroid function in this case.

Date	FT3 (pmol/L)	FT4 (pmol/L)	T3 (nmol/L)	T4 (nmol/L)	TSH (mIU/L)	TPOAB (IU/Ml)	TGAB (IU/mL)
August 17, 2015*	6.39	15.65	2.47	80.72	28.12	149.4	568.5
November 10, 2015	7.00	29.64	2.34	175.4	0.508	129.1	558.8
March 1, 2016	6.22	21.4	2.37	146.6	0.477	48.69	586.4
June 27, 2016	6.31	27.49	2.91	173.8	0.575	28.76	366.7
July 18, 2016	4.27	14.63	1.62	120	14.09	35.94	341.7
September 7, 2016	4.06	16.16	1.60	127.8	23	15.59	172.8
November 1, 2016	6.40	27.09	2.48	173.2	0.059	20.15	153.1
September 3, 2017	5.53	28.81	1.94	174.9	0.041	<5	42.01
March 28, 2018	5.92	21.19	2.18	117.6	1.11	19.16	78.57
December 30, 2018	5.89	22.99	2.34	141.7	0.23	22.19	45.35

Reference value: FT3: 3.1–6.8 pmo/L, FT4: 12–22 pmo/L, T3: 1.3–3.1 nmo/L, T4: 62–164 nmo/L, TSH: 0.270–4.20 mIU/L, TPOAB: 0–115 IU/mL, TGAB: 0–34 IU/mL.

FT3 = free triiodothyronine, FT4 = free thyroxine, T3 = triiodothyronine, T4 = thyroxine, TGAB = anti-thyroglobulin antibodies, TPOAB = thyroid peroxidase antibody, TSH = thyroid stimulating hormone.

* Treatment with Euthyrox began.

### 2.7. Follow-up after treatment

From 2015 to December 30, 2018, the children returned to the hospital regularly for review (results shown in Tables 1–4). The current children’s spirit, appetite significantly better, red-faced, normal limb activity, physical development (Fig. 1), and intelligence were close to those of normal children of the same age. The patient was asked to continue levothyroxine treatment; regularly review thyroid function, routine blood tests, and muscle enzymes; and observe changes in these indicators.

## 3. Discussion

This is the first case report of childhood CH combined with alpha thalassemia, with anemia and muscle damage as the main manifestations in China. This patient did not have typical symptoms of CH, such as special facial features, remarkable intelligence, and poor growth and development. Instead, the main manifestations are anemia and muscle damage. At the same time, because the child had alpha thalassemia, the number of cases in which no diagnosis had been made in several hospitals over several years. CH can affect all body systems, and has a significant impact on children’s intelligence and physical development. Therefore, alpha thalassemia is an autosomal recessive genetic disease, which is a hemolytic anemia caused by partial or complete inhibition of alpha globin chain synthesis due to deletion or defect of the alpha globin gene.^[9]^ The genotyping of the child and his father in this case suggests that they have static α-thalassemia. Because static α-thalassemia has only 1 α gene missing, pathophysiological changes are very slight and cannot be treated.^[10]^ The changes in anemia in children were analyzed (Table 2). First, the children had positive cytochrome anemia, and the worst case was moderate anemia. After 2 blood transfusions, the hemoglobin level only temporarily increased until CH was diagnosed and administered to the levothyroid gland. After oral treatment with plain tablets, routine blood tests were performed several times over the years, and the hemoglobin level increased to normal. Anemia is mainly caused by hypothyroidism. Anemia caused by hypothyroidism is mostly mild to moderate, with positive cytochrome. The main mechanisms may be as follows: ① lack of thyroxine reduces erythropoietin and inhibits hematopoietic function.^[11]^ ② Anti-red blood cell antibodies can be produced in patients with hypothyroidism, which shortens their lifespan of red blood cells. ③ Patients with hypothyroidism can produce anti-gastric parietal cells and anti-intrinsic factor antibodies, which can cause the intestinal absorption of folic acid and folic acid deficiency.^[12]^ ④ Patients with hypothyroidism often lose appetite and decrease gastric acid secretion, resulting in insufficient gastrointestinal absorption of iron, folic acid, and vitamin B12 and insufficient HB and RBC synthesis.^[13]^

CH can affect all body systems and striated muscles can cause hypothyroid muscle damage. The reported muscle damage caused by CH includes hypothyroid myopathy, hypothyroid heart disease, rhabdomyolysis, and asymptomatic or mild symptoms of hypercreatine kinaseaemia.^[14]^ Hypothyroid muscle damage has the following characteristics: (1) generally, it occurs in patients with a long disease course, severe disease, or long-term failure to receive effective treatment. Its characteristic manifestations include elevated serum muscle enzyme and CK levels. The most significant. Clinical manifestations of hypothyroid myopathy include proximal limb weakness, myalgia, muscle cramps, muscle rigidity, masticatory muscle weakness, and pseudohypertrophy. The clinical manifestations of hypothyroid heart disease include chest tightness, electrocardiogram showing ST-T changes, bradycardia, conduction block, and echocardiogram showing pericardial effusion and left ventricular enlargement. Rhabdomyolysis is characterized by a significant increase in CK levels accompanied by obvious limb pain, numbness (combined with bone compartment syndrome), changes in urine color (brown urine), and acute renal failure in severe cases. Hypothyroid muscle damage had a positive effect on thyroxine replacement therapy.^[15]^ (2) Hypothyroid muscle damage can be combined with other diseases caused by hypothyroidism, such as hyperlipidemia, anemia, electrolyte disturbances, hypertension, renal insufficiency, and obstructive ventilation dysfunction, etc.^[16]^ Combined with Table 3, it can be found that the serum muscle enzyme spectrum of this patient was increased, and CK was significantly increased, accompanied by decreased activity tolerance, rigid gastrocnemius of both lower limbs, and color Doppler ultrasound, suggesting a small amount of pericardial effusion and muscles of both lower limbs. The electrogram was abnormal, and the above symptoms were relieved by treatment with levothyroxine sodium, which was consistent with the characteristics of hypothyroid muscle damage. The parents of the child reported that the child had normal growth and development before the age of 1 year and that her height and weight increased slowly after 1. After taking levothyroxine sodium, height and weight gradually approached those of normal children of the same age (Table 1). At the same time, anemia and elevation of muscle enzymes gradually improved after taking levothyroxine sodium, which were close to the normal values, as shown in Tables 2 and 3.

## 4. Strengths and limitations

Strengths: Muscle injury, developmental abnormalities, and anemia are considered to be related to hypothyroidism. Therefore, treatment with levothyroxine significantly improved the symptoms related to muscle injury, developmental abnormalities, and anemia.

Limitations: The patient had a long medical history and presented symptoms and signs of hypothyroidism. However, the diagnosis and treatment of the patient were delayed owing to the failure of the early thyroid function examination. Patient indicators of systemic immune diseases were not measured. Therefore, hypothyroidism caused by abnormal immune function cannot be completely excluded.

## 5. Conclusions

This case suggests that patients with anemia and elevated muscle enzyme levels should be routinely tested for thyroid function to facilitate early detection and treatment and avoid adverse consequences. Analyzing and studying this case is of great significance for reducing missed and misdiagnosed CH, especially in patients with CH combined with alpha thalassemia.

## Acknowledgments

This work was supported by the Scientific Research Fund Project of the Yunnan Provincial Department of Education (grant no. 2021J0232) and the Key Science and Technology Support Project of the Science and Technology Department of Dali Bai Autonomous Prefecture (grant no. D2021NA07).

## Author contributions

**Conceptualization:** Ying Zhang, Xiao-jun Wang, Ming-wei Liu.

**Data curation:** Xuan Li, Xiao-jun Wang, Ju-pin Yang, Yu-ying Dong, Jin-peng Yu, Ming-wei Liu.

**Formal analysis:** Ying Zhang, Ju-mei Li, Yu Wen.

**Funding acquisition:** Xuan Li, Xiao-jun Wang, Ju-pin Yang, Yu-ying Dong, Yu Wen, Ming-wei Liu.

**Investigation:** Ying Zhang, Xuan Li, Ju-mei Li, Wen-qian Yuan, Jin-peng Yu, Yu Wen, Ming-wei Liu.

**Methodology:** Xuan Li, Ju-pin Yang, Yu-ying Dong.

**Project administration:** Ying Zhang, Xuan Li, Xiao-jun Wang, Ju-mei Li, Jin-peng Yu, Yu Wen, Ming-wei Liu.

**Resources:** Ju-pin Yang, Wen-qian Yuan, Jin-peng Yu.

**Software:** Ying Zhang, Xiao-jun Wang, Ju-mei Li, Yu-ying Dong, Yu Wen.

**Supervision:** Xuan Li, Xiao-jun Wang, Wen-qian Yuan, Yu-ying Dong, Ming-wei Liu.

**Validation:** Ying Zhang, Xuan Li, Xiao-jun Wang, Ju-pin Yang, Ju-mei Li, Wen-qian Yuan.

**Visualization:** Xuan Li, Ju-pin Yang, Ju-mei Li, Yu-ying Dong, Ming-wei Liu.

**Writing – original draft:** Ying Zhang, Xuan Li, Xiao-jun Wang, Ju-mei Li, Wen-qian Yuan, Yu-ying Dong, Ming-wei Liu.

**Writing – review & editing:** Ying Zhang, Xuan Li, Xiao-jun Wang, Ju-mei Li, Wen-qian Yuan, Yu-ying Dong, Jin-peng Yu, Ming-wei Liu.
